# Supplementary material for: The Study of Protein–Cyclitol Interactions
Source: Int J Mol Sci. 2022 Mar 9;23(6):2940. doi: 10.3390/ijms23062940 (PMC8952220; doi:10.3390/ijms23062940)
Supplement: Supplementary file 1 [file ijms-23-02940-s001.zip › ijms-1586445-supplementary.pdf]

Supplementary Materials

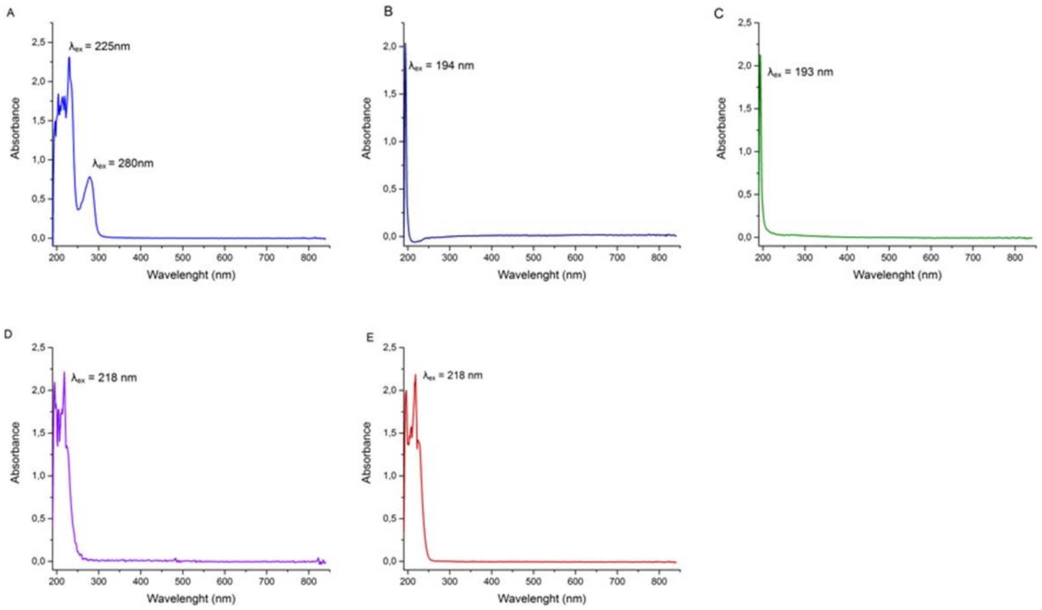

Figure S1. UV-VIS spectra of: A) BSA (1.0 mg/mL); B) D-sorbitol (3.01×10^{-2} M); C) Adonitol (3.01×10^{-2} M); D) Shikimic acid (3.01×10^{-2} M); E) D-(−)-quinic acid (3.01×10^{-2} M).

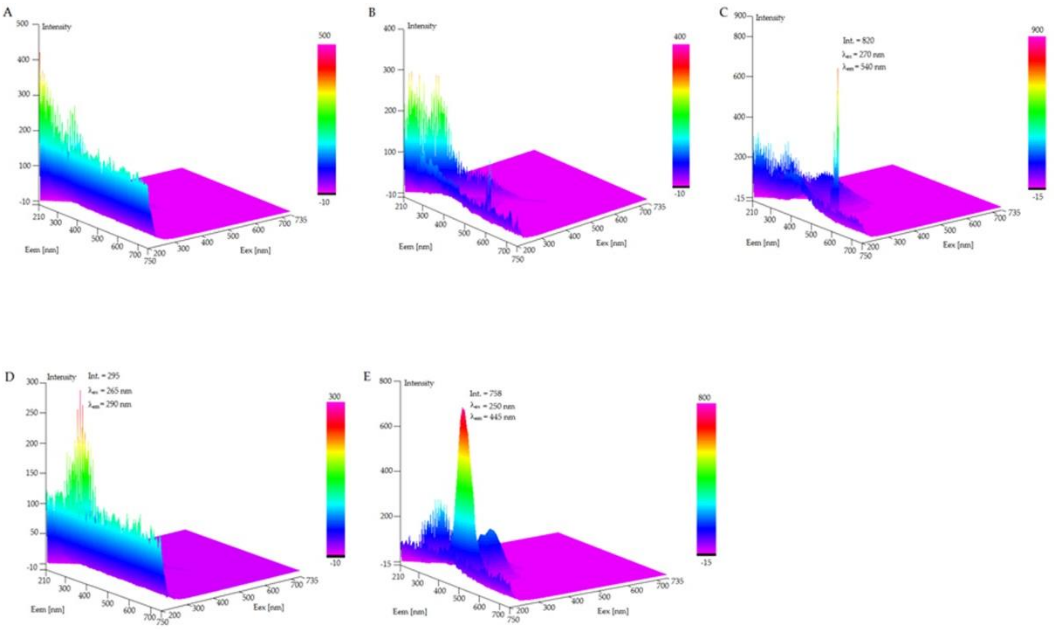

Figure S2. 3D-fluorescence spectra of : A) water; B) D-sorbitol (3.01×10^{-2} M); C) Adonitol (3.01×10^{-2} M); D) Shikimic acid (3.01×10^{-2} M); E) D-(−)-quinic acid (3.01×10^{-2} M).

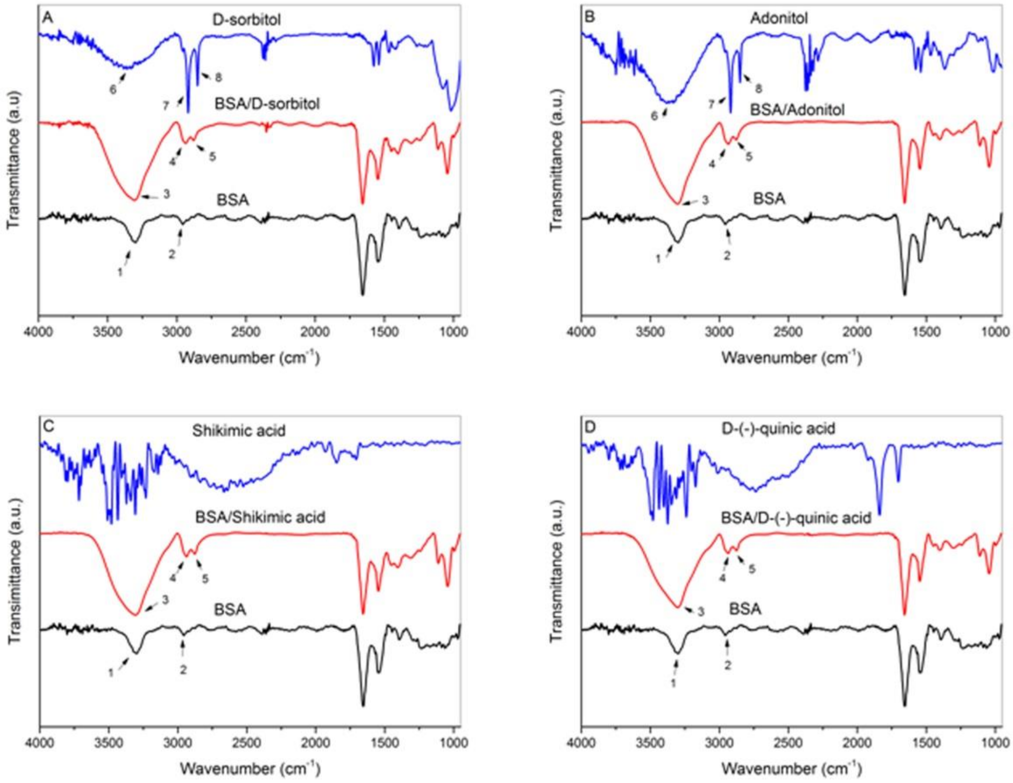

Figure S3. FTIR spectra of BSA, cyclitols and their complexes (at 1:10 molar ratio) in the range of 4000-950 cm^{-1}: A) D-sorbitol (1 – 3300 cm^{-1}, 2 – 2956 cm^{-1}; 3 – 3302 cm^{-1}; 4 – 2936 cm^{-1}; 5 – 2878 cm^{-1}; 6 – 3360 cm^{-1}; 7 – 2918 cm^{-1}; 8 – 2850 cm^{-1}); B) adonitol (1 – 3300 cm^{-1}, 2 – 2956 cm^{-1}; 3 – 3302 cm^{-1}; 4 – 2934 cm^{-1}; 5 – 2876 cm^{-1}; 6 – 3384 cm^{-1}; 7 – 2918 cm^{-1}; 8 – 2850 cm^{-1}); C) shikimic acid (1 – 3300 cm^{-1}, 2 – 2956 cm^{-1}; 3 – 3308 cm^{-1}; 4 – 2936 cm^{-1}; 5 – 2878 cm^{-1}); D) D-(–)-quinic acid (1 – 3300 cm^{-1}, 2 – 2956 cm^{-1}; 3 – 3302 cm^{-1}; 4 – 2936 cm^{-1}; 5 – 2876 cm^{-1}).

Table S1. Detailed list of captioned wavenumbers of **Figure 6**.

A) D-sorbitol	
**Number of peak**	**Wavenumber [cm^{-1}]**
1	1654
2	1568
3	1548
4	1492
5	1446
6	1392
7	1356
8	1294
9	1272
10	1238
11	1122
12	1088
13	1062
14	1000
15	972
16	1656
17	1546
18	1490

19	1448
20	1400
21	1304
22	1246
23	1100
24	1044
25	998
26	1576
27	1540
28	1466
29	1418
30	1264
31	1076
32	1018
**B) Adonitol**	
**Number of peak**	**Wavenumber [cm^{-1}]**
1	1654
2	1568
3	1548
4	1492
5	1446
6	1392
7	1356
8	1294
9	1272
10	1238
11	1122
12	1088
13	1062
14	1000
15	972
16	1656
17	1546
18	1448
19	1400
20	1304
21	1242
22	1122
23	1044
24	994
25	1576
26	1540
27	1466
28	1410
29	1368
30	1126
31	1092
32	1012
**C) Shikimic acid**	
**Number of peak**	**Wavenumber [cm^{-1}]**
1	1654
2	1568

3	1548
4	1492
5	1446
6	1392
7	1356
8	1294
9	1272
10	1238
11	1122
12	1088
13	1062
14	1000
15	972
16	1656
17	1546
18	1454
19	1406
20	1312
21	1242
22	1112
23	1044
24	994
25	1930
26	1850
27	1732
28	1706
**D) D-(−)-quinic acid**	
**Number of peak**	**Wavenumber [cm^{-1}]**
1	1654
2	1568
3	1548
4	1492
5	1446
6	1392
7	1356
8	1294
9	1272
10	1238
11	1122
12	1088
13	1062
14	1000
15	972
16	1658
17	1548
18	1448
19	1402
20	1302
21	1242
22	1112
23	1044
24	996

25	1922
26	1840
27	1702

Table S2. Detailed list of captioned wavenumbers of **Figure 7**.

**A) D-sorbitol**	
**Number of peak**	**Wavenumber [cm^{-1}]**
1	1568
2	1511
3	1417
4	1309
5	1096
6	976
7	1604
8	1568
9	1504
10	1456
11	1412
12	1349
13	1307
14	1234
15	1178
16	1095
17	1045
18	987
19	1451
20	1255
21	1127
22	1050
23	940
**B) Adonitol**	
**Number of peak**	**Wavenumber [cm^{-1}]**
1	1568
2	1511
3	1417
4	1309
5	1096
6	976
7	1569
8	1509
9	1414
10	1309
11	1094
12	1451

13	1431
14	1376
15	1340
16	1268
17	1132
18	1064
19	1034
20	946
**C) Shikimic acid**	
**Number of peak**	**Wavenumber [cm^{-1}]**
1	1568
2	1511
3	1417
4	1309
5	1096
6	976
7	1568
8	1509
9	1414
10	1309
11	1095
12	1645
13	1441
14	1385
15	1350
16	1269
17	1095
18	1035
**D) D-(−)-quinic acid**	
**Number of peak**	**Wavenumber [cm^{-1}]**
1	1568
2	1511
3	1417
4	1309
5	1096
6	976
7	1568
8	1508
9	1415
10	1308
11	1095
12	976
13	1679
14	1513
15	1457

16	1422
17	1098
18	976
